# Family healing: Contextual interventions in perinatal clinical practice

**DOI:** 10.1192/j.eurpsy.2021.79

**Published:** 2021-08-13

**Authors:** T. Kurimay, T. Fenyves, A. Pelikan

**Affiliations:** Buda Family Centre Mh Centre Department Of Psychiatry, Teaching Dep. Of Semmelweis University, North Centre Buda New Saint John Hospital and Oupatient Clinic, Budapest, Hungary

## Abstract

**Abstract Body:**

In perinatal clinical practice (PCP), the focus of care has shifted from the mother and then the baby-mother dyad to the emphasis on the role of fathers. Individual and therapeutic interventions are multimodal, and in almost all cases interdisciplinary cooperation is assumed. The preferred therapeutic methods for perinatal mental disorders are psychological and psychotherapeutic interventions. Through the life-course model, the central, therapeutic-conceptual role of the family can be understood, which - in clinical practice - reflects the need for “think-family” in psychiatric care. Hence there is a growing need for evidence-based family-interventions. Parental mental health disorders may have an impact on family functioning and partner relationship, as well as parent-child interactions, the quality of attachment and relationship with the child. Even though we have an increasing number of evidence regarding the aims and effectiveness of family interventions, additional evidence is needed to determine what interventions and modalities are effective in the perinatal period. And we also need information when these interventions are contraindicated and regarding their risk. It is conceivable that there is not much difference between the efficacy of family intervention methods used in other indications and the perinatal application of the same methods. We have gathered evidence primarily on perinatal depression, which opens the path of family interventions in other disorders. When thinking in the family as a general framework, it should be filled with evidence-based quantitative and qualitative anchors. The conceptual framework can be based on systems- and network theory. The presentation is illustrated with clinical vignettes.

**Disclosure:**

No significant relationships.

